# 3-Chloro-*N*-[*N*-(furan-2-carbon­yl)hydrazinocarbo­thio­yl]benzamide

**DOI:** 10.1107/S1600536813025440

**Published:** 2013-09-21

**Authors:** Bohari M Yamin, Diyana Yusof, Siti Aishah Hasbullah

**Affiliations:** a School of Chemical Sciences and Food Technology, Universiti Kebangsaan Malaysia, 43600 Bangi, Selangor, Malaysia

## Abstract

In the title compound C_13_H_10_ClN_3_O_3_S, the benzoyl group maintains its *trans* conformation against the thiono group about the C—N bond and the intra­molecular hydrogen bond between the benzoyl O atom and thio­amide H atom. In the crystal, N—H⋯O and C—H⋯O hydrogen bonds link the mol­ecules, forming chains along the *b*-axis direction. In addition, C—H⋯π inter­actions occur between a phenyl H atom and the furan ring.

## Related literature

For bond-lengths data, see: Allen *et al.* (1987 ▶) and for a description of the Cambridge Structural Database, see: Allen (2002 ▶). For related structures of thio­urea derivatives, see: Yamin & Yusof (2003 ▶); Yusof *et al.* (2003 ▶); Ali *et al.* (2004 ▶); Venkatachalam *et al.* (2004 ▶); Saeed *et al.* (2011 ▶); Wilson *et al.* (2010 ▶).
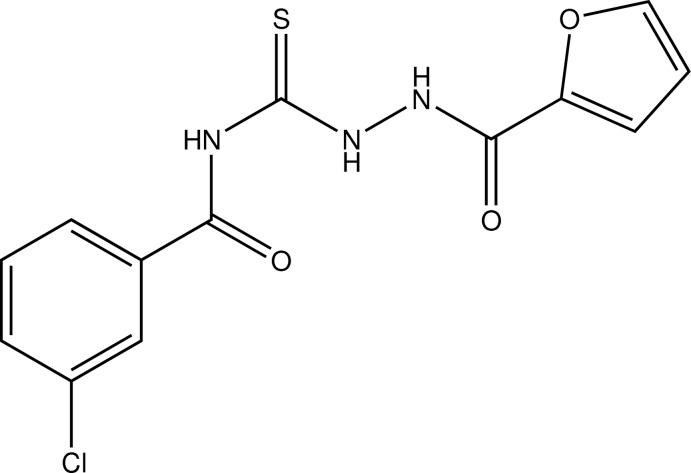


## Experimental

### 

#### Crystal data

C_13_H_10_ClN_3_O_3_S
*M* *_r_* = 323.75
Orthorhombic, 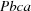

*a* = 7.286 (4) Å
*b* = 15.148 (8) Å
*c* = 25.840 (14) Å
*V* = 2852 (3) Å^3^
*Z* = 8
Mo *K*α radiation
μ = 0.43 mm^−1^
*T* = 298 K
0.50 × 0.49 × 0.12 mm

#### Data collection

Bruker SMART APEX CCD area-detector diffractometer
Absorption correction: multi-scan (*SADABS*; Bruker, 2000 ▶) *T*_min_ = 0.815, *T*_max_ = 0.951
15224 measured reflections
2507 independent reflections
1723 reflections with *I* > 2σ(*I*)
*R*_int_ = 0.082

#### Refinement

*R*[*F* ^2^ > 2σ(*F* ^2^)] = 0.045
*wR*(*F* ^2^) = 0.117
*S* = 1.02
2507 reflections
190 parameters
H-atom parameters constrained
Δρ_max_ = 0.24 e Å^−3^
Δρ_min_ = −0.19 e Å^−3^

### 

Data collection: *SMART* (Bruker, 2000 ▶); cell refinement: *SAINT* (Bruker, 2000 ▶); data reduction: *SAINT*; program(s) used to solve structure: *SHELXTL* (Sheldrick, 2008 ▶); program(s) used to refine structure: *SHELXTL*; molecular graphics: *SHELXTL*; software used to prepare material for publication: *SHELXTL* and *PLATON* (Spek, 2009 ▶).

## Supplementary Material

Crystal structure: contains datablock(s) global, I. DOI: 10.1107/S1600536813025440/bg2516sup1.cif

Structure factors: contains datablock(s) I. DOI: 10.1107/S1600536813025440/bg2516Isup2.hkl

Additional supplementary materials:  crystallographic information; 3D view; checkCIF report

## Figures and Tables

**Table 1:** Hydrogen-bond geometry (Å, °) *Cg* is the centroid of the furan ring.

*D*—H⋯*A*	*D*—H	H⋯*A*	*D*⋯*A*	*D*—H⋯*A*
N2—H2*A*⋯O1	0.86	1.92	2.574 (3)	132
N1—H1*A*⋯O2^i^	0.86	2.25	3.094 (3)	167
C5—H5*A*⋯O2^i^	0.93	2.32	3.093 (4)	140
C13—H13*A*⋯*Cg* ^ii^	0.93	2.83	3.516 (4)	132

Symmetry codes: (i) 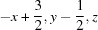; (ii) 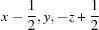.

**Fig. 1.**
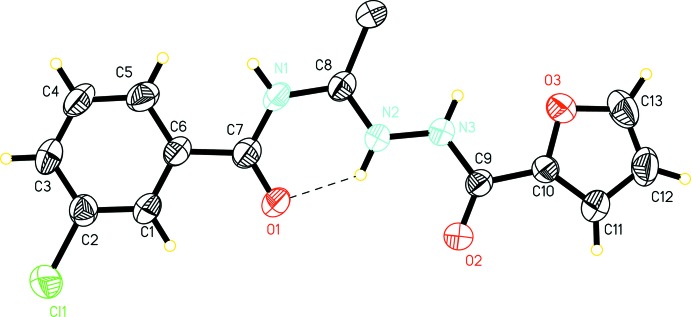
The molecular structure of (I), with displacement ellipsods drawn at the 50% probability level. The dashed line indicates intramolecular hydrogen bond.

**Fig. 2.**
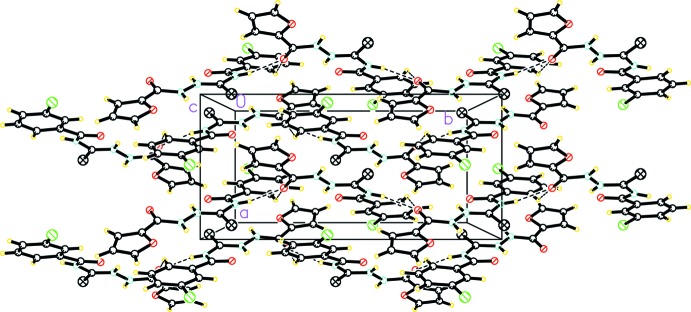
Molecular packing of (I) viewed down the c-axis. Dashed lines indicate intermolecular hydrogen bonds.

## supplementary crystallographic information

### 1. Comment

Structural studies of thiourea derivatives have received much attention for the last ten years or so. Their applications in biological activities (Venkatachalam *et al.*, 2004; Saeed *et al.*, 2011) and sensor development as ionophore (Wilson *et al.*, 2010) are important. However, the derivatives consisting of hydrazine are relatively less frequent. On the other hand, the presence of the hydrazinothiocarbonyl group could make the thiourea a good candidate for tridentate chelation with metals. The title compound (I) is similar to N-[N-(furan-2-carbonyl)hydrazinothio carbonyl)benzamide (Yamin & Yusof, 2003) except in the chlorine atom attached at position-3 of the benzene ring (Fig.1). Bond lengths and angles in (I) are in normal ranges (Allen *et al.*, 1987; Allen, 2002) and comparable to those in the analogue N-(N-benzoylhydrazinocarbo thioyl)benzamide (Yusof *et al.* 2003) and 2-chloro-N-(N-(4-chlorobenzoyl)hydrazinecarbonothioyl)benzamide (Ali *et al.*, 2004). The whole molecule looks nearly planar, with small dihedral angles between the central thiourea moiety N1/C8/S1/N2/N3 with the chlorobenzene, Cl1/(C1-C7) and carbonylfuran O2/O3/(C9-C13) ( 6.07 (9) and 4.82 (11)° respectively). The dihedral angle between the chlorobenzene and carbonylfuran is 10.1 (11)°. All the fragments are planar with maximum deviation from their least square plane for C9 atom of the carbonylfuran (0.030 (3)Å ). There is a significant N2–H2A···O1 intramolecular hydrogen bond (Table 2) which is usually present in any trans carbonoylthiourea (with respect to the position of the carbonoyl group against the thiono about the C8-N1 bond). In the crystal structure, the molecules are linked by N–H··O and C–H···O intermolecular hydrogen bonds (see Table 2) to form one-dimensional chains along the b-axis (Fig.2). In addition, there is a C13–H13A···Cg^ii^ interaction (Cg, the centroid of the furan ring O3,C10,C11,C12,C13; (ii): -1/2+x,y,1/2-z) with a H···Cg^ii^ distance = 2.830Å and a C-H..Cg^ii^ angle = 132°.

### 2. Experimental

An acetone (30 ml) solution of tetrahydrofuran-2-carboxamide (0.18 g, 2 mmol) was added into a round-bottom flask containing 3-chlorobenzoyl isothiocyanate (0.58 g,2 mmol). The mixture was refluxed for 3h. After cooling, the solution was filtered off and the filtrate was left to evaporate at room temperature. The solid formed was washed with water and cold ethanol. Crystals suitable for X-ray study were obtained by recrystallization from DMSO.

### 3. Refinement

Crystal data, data collection and structure refinement details are summarized in Table 1. All H atoms attached to C and N atoms were fixed geometrically and treated as riding with C—H= 0.93-0.97Å and N–H = 0.86Å with U_iso_(H)= 1.2U_eq_(C and N).

### Crystal data

**Table:**

C_13_H_10_ClN_3_O_3_S	*F*(000) = 1328
*M**_r_* = 323.75	*D*_x_ = 1.508 Mg m^−^^3^
Orthorhombic, *P**b**c**a*	Mo *K*α radiation, λ = 0.71073 Å
Hall symbol: -P 2ac 2ab	Cell parameters from 1801 reflections
*a* = 7.286 (4) Å	θ = 1.5–25.0°
*b* = 15.148 (8) Å	µ = 0.43 mm^−^^1^
*c* = 25.840 (14) Å	*T* = 298 K
*V* = 2852 (3) Å^3^	Block, colourless
*Z* = 8	0.50 × 0.49 × 0.12 mm

### Data collection

**Table:**

Bruker SMART APEX CCD area-detector diffractometer	2507 independent reflections
Radiation source: fine-focus sealed tube	1723 reflections with *I* > 2σ(*I*)
Graphite monochromator	*R*_int_ = 0.082
Detector resolution: 83.66 pixels mm^-1^	θ_max_ = 25.0°, θ_min_ = 1.6°
ω scan	*h* = −8→8
Absorption correction: multi-scan (*SADABS*; Bruker, 2000)	*k* = −18→18
*T*_min_ = 0.815, *T*_max_ = 0.951	*l* = −30→20
15224 measured reflections	

### Refinement

**Table:**

Refinement on *F*^2^	Primary atom site location: structure-invariant direct methods
Least-squares matrix: full	Secondary atom site location: difference Fourier map
*R*[*F*^2^ > 2σ(*F*^2^)] = 0.045	Hydrogen site location: inferred from neighbouring sites
*wR*(*F*^2^) = 0.117	H-atom parameters constrained
*S* = 1.02	*w* = 1/[σ^2^(*F*_o_^2^) + (0.*P*)^2^ + 1.0899*P*] where *P* = (*F*_o_^2^ + 2*F*_c_^2^)/3
2507 reflections	(Δ/σ)_max_ < 0.001
190 parameters	Δρ_max_ = 0.24 e Å^−^^3^
0 restraints	Δρ_min_ = −0.19 e Å^−^^3^

### Special details

**Table:**

Geometry. All esds (except the esd in the dihedral angle between two l.s. planes) are estimated using the full covariance matrix. The cell esds are taken into account individually in the estimation of esds in distances, angles and torsion angles; correlations between esds in cell parameters are only used when they are defined by crystal symmetry. An approximate (isotropic) treatment of cell esds is used for estimating esds involving l.s. planes.
Refinement. Refinement of F^2^ against ALL reflections. The weighted R-factor wR and goodness of fit S are based on F^2^, conventional R-factors R are based on F, with F set to zero for negative F^2^. The threshold expression of F^2^ > 2sigma(F^2^) is used only for calculating R-factors(gt) etc. and is not relevant to the choice of reflections for refinement. R-factors based on F^2^ are statistically about twice as large as those based on F, and R- factors based on ALL data will be even larger.

### Fractional atomic coordinates and isotropic or equivalent isotropic displacement parameters (Å^2^)

**Table:**

	*x*	*y*	*z*	*U*_iso_*/*U*_eq_	
Cl1	0.98435 (14)	−0.08651 (5)	−0.18005 (3)	0.0735 (3)	
O1	0.7448 (3)	0.04610 (12)	−0.00613 (7)	0.0591 (6)	
O2	0.6645 (3)	0.25070 (11)	0.08567 (7)	0.0509 (5)	
O3	0.4281 (3)	0.21465 (12)	0.20364 (7)	0.0509 (5)	
N1	0.7094 (3)	−0.06226 (13)	0.05323 (8)	0.0436 (6)	
H1A	0.7296	−0.1173	0.0592	0.052*	
N2	0.6422 (3)	0.07378 (13)	0.08781 (8)	0.0419 (6)	
H2A	0.6885	0.0964	0.0602	0.050*	
N3	0.5717 (3)	0.12697 (13)	0.12567 (9)	0.0461 (6)	
H3A	0.5170	0.1039	0.1519	0.055*	
C1	0.8615 (4)	−0.06793 (17)	−0.08220 (11)	0.0436 (7)	
H1B	0.8702	−0.0074	−0.0878	0.052*	
C2	0.9095 (4)	−0.12615 (18)	−0.12060 (11)	0.0441 (7)	
C3	0.8981 (4)	−0.21559 (17)	−0.11338 (12)	0.0474 (7)	
H3B	0.9332	−0.2543	−0.1395	0.057*	
C4	0.8341 (5)	−0.24695 (18)	−0.06697 (12)	0.0543 (8)	
H4A	0.8249	−0.3075	−0.0618	0.065*	
C5	0.7834 (4)	−0.19013 (17)	−0.02794 (11)	0.0510 (8)	
H5A	0.7379	−0.2123	0.0031	0.061*	
C6	0.8002 (4)	−0.09970 (16)	−0.03502 (10)	0.0388 (6)	
C7	0.7507 (4)	−0.03252 (17)	0.00457 (10)	0.0418 (7)	
C8	0.6385 (4)	−0.01326 (16)	0.09412 (10)	0.0396 (6)	
C9	0.5875 (4)	0.21534 (15)	0.12203 (10)	0.0356 (6)	
C10	0.5023 (4)	0.26306 (16)	0.16468 (9)	0.0360 (6)	
C11	0.4757 (4)	0.34892 (18)	0.17338 (12)	0.0501 (8)	
H11A	0.5155	0.3954	0.1527	0.060*	
C12	0.3764 (5)	0.3558 (2)	0.21962 (12)	0.0593 (9)	
H12A	0.3359	0.4075	0.2353	0.071*	
C13	0.3516 (5)	0.2743 (2)	0.23661 (12)	0.0613 (9)	
H13A	0.2902	0.2598	0.2670	0.074*	
S1	0.55517 (13)	−0.06141 (5)	0.14664 (3)	0.0566 (3)	

### Atomic displacement parameters (Å^2^)

**Table:**

	*U*^11^	*U*^22^	*U*^33^	*U*^12^	*U*^13^	*U*^23^
Cl1	0.1084 (8)	0.0531 (5)	0.0589 (6)	−0.0122 (5)	0.0308 (5)	−0.0102 (4)
O1	0.1014 (18)	0.0309 (11)	0.0451 (12)	0.0079 (10)	0.0097 (11)	0.0008 (9)
O2	0.0729 (15)	0.0372 (10)	0.0427 (11)	−0.0040 (9)	0.0154 (10)	0.0013 (9)
O3	0.0632 (14)	0.0457 (11)	0.0437 (12)	0.0032 (9)	0.0137 (10)	0.0024 (9)
N1	0.0614 (16)	0.0247 (11)	0.0448 (14)	0.0032 (10)	−0.0005 (11)	−0.0022 (10)
N2	0.0572 (15)	0.0303 (11)	0.0381 (13)	0.0018 (10)	0.0081 (11)	−0.0017 (10)
N3	0.0648 (17)	0.0317 (12)	0.0416 (13)	0.0010 (11)	0.0137 (12)	−0.0021 (10)
C1	0.0508 (18)	0.0302 (13)	0.0497 (18)	−0.0015 (12)	−0.0007 (14)	−0.0064 (12)
C2	0.0432 (17)	0.0421 (15)	0.0469 (17)	−0.0036 (13)	0.0029 (13)	−0.0076 (13)
C3	0.0535 (19)	0.0373 (15)	0.0512 (18)	0.0082 (13)	−0.0081 (15)	−0.0126 (13)
C4	0.080 (2)	0.0290 (14)	0.0535 (18)	0.0094 (14)	−0.0144 (16)	−0.0050 (13)
C5	0.076 (2)	0.0367 (16)	0.0406 (17)	0.0027 (14)	−0.0108 (15)	0.0022 (12)
C6	0.0464 (17)	0.0300 (14)	0.0401 (15)	0.0044 (11)	−0.0074 (13)	−0.0023 (11)
C7	0.0494 (17)	0.0344 (15)	0.0417 (17)	0.0026 (12)	−0.0064 (13)	−0.0021 (12)
C8	0.0437 (16)	0.0315 (14)	0.0436 (16)	0.0001 (12)	−0.0046 (13)	−0.0033 (12)
C9	0.0396 (16)	0.0306 (14)	0.0365 (15)	0.0007 (11)	−0.0035 (12)	0.0001 (11)
C10	0.0396 (16)	0.0354 (13)	0.0329 (14)	−0.0010 (12)	−0.0013 (12)	0.0003 (11)
C11	0.065 (2)	0.0369 (15)	0.0489 (18)	0.0036 (14)	0.0038 (15)	−0.0045 (13)
C12	0.072 (2)	0.0511 (19)	0.055 (2)	0.0136 (16)	0.0045 (17)	−0.0165 (16)
C13	0.062 (2)	0.076 (2)	0.0457 (18)	0.0112 (18)	0.0154 (16)	−0.0076 (16)
S1	0.0856 (6)	0.0331 (4)	0.0513 (5)	−0.0044 (4)	0.0144 (4)	0.0026 (3)

### Geometric parameters (Å, º)

**Table:**

Cl1—C2	1.737 (3)	C2—C3	1.370 (4)
O1—C7	1.223 (3)	C3—C4	1.372 (4)
O2—C9	1.219 (3)	C3—H3B	0.9300
O3—C10	1.358 (3)	C4—C5	1.376 (4)
O3—C13	1.362 (3)	C4—H4A	0.9300
N1—C7	1.369 (3)	C5—C6	1.388 (4)
N1—C8	1.391 (3)	C5—H5A	0.9300
N1—H1A	0.8600	C6—C7	1.488 (4)
N2—C8	1.329 (3)	C8—S1	1.656 (3)
N2—N3	1.368 (3)	C9—C10	1.457 (3)
N2—H2A	0.8600	C10—C11	1.334 (4)
N3—C9	1.347 (3)	C11—C12	1.401 (4)
N3—H3A	0.8600	C11—H11A	0.9300
C1—C2	1.373 (4)	C12—C13	1.322 (4)
C1—C6	1.385 (4)	C12—H12A	0.9300
C1—H1B	0.9300	C13—H13A	0.9300
			
C10—O3—C13	105.6 (2)	C1—C6—C5	119.2 (2)
C7—N1—C8	127.1 (2)	C1—C6—C7	116.5 (2)
C7—N1—H1A	116.4	C5—C6—C7	124.3 (3)
C8—N1—H1A	116.4	O1—C7—N1	121.4 (2)
C8—N2—N3	119.3 (2)	O1—C7—C6	121.3 (3)
C8—N2—H2A	120.4	N1—C7—C6	117.4 (2)
N3—N2—H2A	120.4	N2—C8—N1	115.4 (2)
C9—N3—N2	120.2 (2)	N2—C8—S1	123.0 (2)
C9—N3—H3A	119.9	N1—C8—S1	121.59 (18)
N2—N3—H3A	119.9	O2—C9—N3	122.0 (2)
C2—C1—C6	119.7 (2)	O2—C9—C10	124.2 (2)
C2—C1—H1B	120.2	N3—C9—C10	113.8 (2)
C6—C1—H1B	120.2	C11—C10—O3	110.1 (2)
C3—C2—C1	121.4 (3)	C11—C10—C9	132.3 (2)
C3—C2—Cl1	118.8 (2)	O3—C10—C9	117.5 (2)
C1—C2—Cl1	119.8 (2)	C10—C11—C12	106.9 (3)
C2—C3—C4	118.8 (3)	C10—C11—H11A	126.5
C2—C3—H3B	120.6	C12—C11—H11A	126.5
C4—C3—H3B	120.6	C13—C12—C11	106.6 (3)
C3—C4—C5	121.0 (3)	C13—C12—H12A	126.7
C3—C4—H4A	119.5	C11—C12—H12A	126.7
C5—C4—H4A	119.5	C12—C13—O3	110.9 (3)
C4—C5—C6	119.8 (3)	C12—C13—H13A	124.6
C4—C5—H5A	120.1	O3—C13—H13A	124.6
C6—C5—H5A	120.1		
			
C8—N2—N3—C9	174.6 (2)	N3—N2—C8—N1	178.7 (2)
C6—C1—C2—C3	0.0 (4)	N3—N2—C8—S1	−1.2 (4)
C6—C1—C2—Cl1	179.4 (2)	C7—N1—C8—N2	−12.8 (4)
C1—C2—C3—C4	1.2 (4)	C7—N1—C8—S1	167.1 (2)
Cl1—C2—C3—C4	−178.2 (2)	N2—N3—C9—O2	−0.3 (4)
C2—C3—C4—C5	−0.6 (5)	N2—N3—C9—C10	178.8 (2)
C3—C4—C5—C6	−1.3 (5)	C13—O3—C10—C11	0.9 (3)
C2—C1—C6—C5	−1.8 (4)	C13—O3—C10—C9	−177.3 (2)
C2—C1—C6—C7	−179.9 (3)	O2—C9—C10—C11	5.0 (5)
C4—C5—C6—C1	2.5 (4)	N3—C9—C10—C11	−174.1 (3)
C4—C5—C6—C7	−179.6 (3)	O2—C9—C10—O3	−177.3 (2)
C8—N1—C7—O1	7.6 (5)	N3—C9—C10—O3	3.6 (3)
C8—N1—C7—C6	−171.5 (2)	O3—C10—C11—C12	−1.2 (3)
C1—C6—C7—O1	8.0 (4)	C9—C10—C11—C12	176.6 (3)
C5—C6—C7—O1	−170.0 (3)	C10—C11—C12—C13	1.1 (4)
C1—C6—C7—N1	−172.9 (2)	C11—C12—C13—O3	−0.5 (4)
C5—C6—C7—N1	9.1 (4)	C10—O3—C13—C12	−0.2 (4)

### Hydrogen-bond geometry (Å, º)

Cg is the centroid of the furan ring.

**Table:**

*D*—H···*A*	*D*—H	H···*A*	*D*···*A*	*D*—H···*A*
N2—H2*A*···O1	0.86	1.92	2.574 (3)	132
N1—H1*A*···O2^i^	0.86	2.25	3.094 (3)	167
C5—H5*A*···O2^i^	0.93	2.32	3.093 (4)	140
C13—H13*A*···*Cg*^ii^	0.93	2.83	3.516 (4)	132

Symmetry codes: (i) −*x*+3/2, *y*−1/2, *z*; (ii) *x*−1/2, *y*, −*z*+1/2.
